# The Impact of Psoriasis Treatment on Depression and Suicide Risk: A Systematic Review

**DOI:** 10.1155/da/6275455

**Published:** 2026-07-10

**Authors:** Behnoosh Shahsavaripoor, Shaghayegh Karami, Ali Sahebi, Mohammad Jafferany

**Affiliations:** ^1^ Department of Psychiatry and Behavioral Health, Nisonger Center, College of Medicine, Ohio State University Wexner Medical Center, Columbus, Ohio, USA, osu.edu; ^2^ Students’ Scientific Research Center (SSRC), Tehran University of Medical Sciences, Tehran, Iran, tums.ac.ir; ^3^ Department of Medical Emergencies and Health in Disasters and Emergencies, Ilam University of Medical Sciences, Ilam, Iran, medilam.ac.ir; ^4^ Department of Psychiatry, Central Michigan University/CMU Medical Education Partners, Saginaw, Michigan, USA, cmich.edu

**Keywords:** biologic therapy, depression, psoriasis, suicidal ideation, suicide

## Abstract

**Background:**

Psoriasis is a long‐lasting inflammatory condition of the skin associated with various comorbidities, including depression and suicidal ideation. Management strategies for psoriasis include symptom alleviation, quality‐of‐life enhancement, and prevention of disease progression. Psoriasis treatments include topical therapies, phototherapy, oral systemic medications, and biologics.

**Objectives:**

In this review, we evaluate the impact of psoriasis treatments on depression and suicidal ideation in affected patients.

**Methods:**

We systematically searched multiple databases, including PubMed, Scopus, and Web of Science, until September 30, 2024, to identify relevant articles. Studies that examined the effects of psoriasis therapies on depression and suicidal thoughts were included. Data on treatment modalities, psychological health outcomes, and psoriasis severity was obtained. Quality evaluation instruments, such as JBI, consolidated standards of reporting trials (CONSORTs), Center for Evidence‐Based Management (CEMBa), and appraisal tool for cross‐sectional studies (AXIS), were used to appraise study quality.

**Results:**

Ten studies met inclusion criteria, predominantly focusing on biologic therapies. Biologics like guselkumab, brodalumab, and bimekizumab have shown notable reductions in depression symptoms, probably through the alleviation of psoriasis severity and the enhancement of quality‐of‐life. Suicidal ideation occurred in some cases, especially with brodalumab; however, a definitive causal relationship was not established.

**Conclusions:**

Treatments for psoriasis, especially biologics, have shown some advantages in reducing depression symptoms as well as relieving skin symptoms. However, cautious monitoring is required due to the possible hazards of suicidal thoughts with certain therapeutic options such as biologics. Addressing the complex issues of psoriasis requires comprehensive therapy that includes dermatologists and mental health specialists.

## 1. Introduction

Psoriasis is a persistent inflammatory condition that can simultaneously impact the skin and joints or both. It is characterized by significant genetic predisposition and autoimmune pathogenic features [1, 2]. This condition affects ~2% of the global population, with variability observed based on skin type [3]. The disease is characterized by a lifelong stigma and imposes both physical and psychological burdens, leading to a significant reduction in the patient’s quality‐of‐life. Psoriasis is presently regarded as a multifactorial disease, with various genetic and environmental factors potentially contributing to its onset or progression [4]. The interaction between genetic and immunological cytokines (IL‐1, IL‐6, and tumor necrosis factor‐alpha) is critical in developing psoriasis [5, 6].

Approximately 75% of individuals with psoriasis present with at least one comorbidity. Examples of diseases include diabetes [7], cardiovascular diseases [8], uveitis [9], inflammatory bowel disease [10], osteoporosis [11], and neurological and psychiatric diseases [5]. Additionally, individuals with psoriasis exhibit a markedly increased prevalence of psychiatric disorders, such as anxiety and depression, in comparison to both the general population and other dermatological patient groups [12, 13]. Psychiatric implications can both arise from and exacerbate psoriasis, potentially creating a vicious cycle of deteriorating physical diseases and mental health disorders [14]. Evidence indicates that depression may exacerbate or initiate psoriasis symptoms [14].

Psoriasis impacts patients’ quality‐of‐life and life expectancy; however, a cure for the disease remains unachieved. Several treatment options exist to mitigate skin manifestations. Topical therapies [15], phototherapy [16], oral systemic therapies [17], and biologic therapies are several proposed therapeutic options for psoriasis [18]. Biologics represent a category of pharmaceuticals consisting of large, complex protein molecules, such as monoclonal antibodies and receptor fusion proteins, that specifically target immune system components implicated in psoriasis’s pathogenesis [19]. Biologics are recommended for treating moderate‐to‐severe psoriasis that has not adequately responded to conventional systemic therapies [20]. Topical therapy is generally the initial treatment for patients with mild or limited‐extent disease. If this is inadequate, the condition is classified as moderate‐to‐severe, prompting the use of phototherapy or conventional systemic therapies [19]. These treatments are associated with several side effects, including gastrointestinal complications, kidney failure, and malignancies. There is an increasing need to understand the pathogenic pathways in psoriasis to discover novel therapeutic strategies that minimize side effects [18]. A diverse team of clinicians with various expertise areas is frequently essential for effectively treating the disease, aiming to optimize outcomes while minimizing side effects [1].

The treatment methods for psoriasis can significantly impact patients, particularly concerning depression levels and the emergence of suicidal thoughts, as noted in various studies. Research in this domain has documented a range of complications and varying outcomes. This study was designed to investigate the impact of these treatment methods on the patients’ depression and suicidal thoughts.

## 2. Materials and Methods

### 2.1. Search Strategy and Study Selection

A systematic review was performed following the preferred reporting items for systematic reviews and meta‐analyses (PRISMA) guidelines [21]. This review was registered in PROSPERO (International Prospective Register of Systematic Reviews): CRD42024622627.

**Figure 1:**
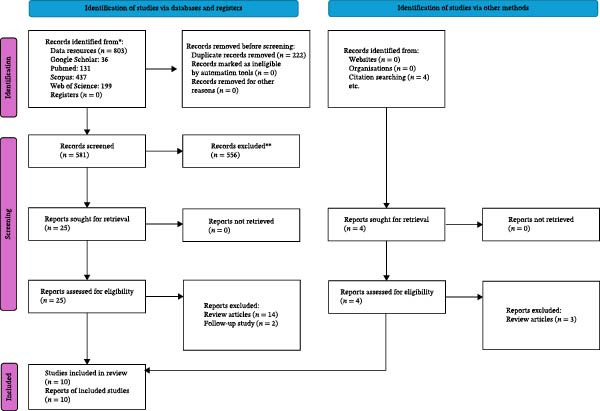
Flow diagram of study selection process for the systematic review, following the PRISMA 2020 guidelines. Note: This diagram outlines the process of study selection for the systematic review, showing the number of records identified, screened, assessed for eligibility, and included in the final analysis. From: Page et al. [22]. The PRISMA 2020 statement: an updated guideline for reporting systematic reviews. BMJ 2021;372:n71. doi: 10.1136/bmj.n71. For more information, visit: http://www.prisma-statement.org/.

For this study, we searched all accessible studies in electronic databases until September 30, 2024. This included PubMed, Scopus, Web of Science, Scopus, and Google Scholar. We did not restrict our search by study type or publication year. Keywords like “Anti‐psoriatic drugs,” “psoriasis treatment,” “depression,” and “suicide ideation” were thoughtfully used to search each database. The complete article selection process for our systematic review can be found in Figure 1.

### 2.2. Eligibility Criteria

Our study utilized a PICO framework (P: Individuals with psoriasis, I: Psoriasis treatment [e.g., topical, systemic, and biologic therapies], C: Not applicable, and O: Incidence and severity of depression and suicide) to address the research objective. The eligibility criteria included any English‐language paper evaluating the impact of psoriatic treatments on depression and suicide.

### 2.3. Screening Process

The records were imported into EndNote.21 software for the study selection process after retrieval from each database and removing duplicates. Two reviewers, A.S. and B.S., conducted independent assessments of the papers through a two‐step process, including title and abstract screening, followed by a full‐text evaluation. In both phases, the third author resolved conflicts and disagreements.

The inclusion criteria consisted of (i) original studies published in English, (ii) studies involving adult patients (≥18 years) with a confirmed diagnosis of psoriasis, and (iii) articles that evaluate the application of a therapeutic method and assess its impact on mental health. Studies involving pediatric patients aged under 18 years and studies lacking pertinent data to our research question were excluded. Furthermore, animal studies, conference abstracts, editorials, commentaries, letters to the editors, book chapters, opinion articles, systematic reviews, meta‐analyses, and review articles were excluded.

### 2.4. Quality Assessment

Different checklists for this systematic review have been used by two independent reviewers (S.K. and B.S.) to evaluate the quality of studies systematically examining outcomes associated with depression or suicidal ideation. For cohort studies, the JBI Checklist was used to assess the quality of included cohort studies [23]. Studies receiving a JBI score above 70% are categorized as high quality, those scoring between 50% and 70% as moderate quality, and those scoring below 50% as low quality. The consolidated standards of reporting trials (CONSORTs) were used for clinical trial research. This instrument has 25 items rated from 0 to 2, with the lowest and highest scores being 0 and 50, respectively. Scores 0–16, 17−33, and 34–50 denoted low, moderate, and good qualities, respectively [24]. The appraisal tool for cross‐sectional studies (AXIS) was used to assess the quality of selected cross‐sectional studies [25]. The instrument had a scoring range of 0–20. Studies with scores over 12 were deemed of moderate or high quality. The Center for Evidence‐Based Management (CEMBa) was used for case studies. This tool comprises 10 sections, each rated from 0 to 1. The 0–2, 3–6, and 7–10 ratings signified poor, moderate, and good qualities, respectively [24]. Conflicts were settled via discussion until a consensus was reached.

### 2.5. Data Extraction Process

Research articles that met the eligibility criteria during the title and abstract screening processes were selected for data extraction during the thorough full‐text evaluation. Two reviewers, B.S. and S.K., conducted the data extraction process utilizing a predesigned data spreadsheet. The following data were retrieved for each study: title, study design, country, publication year, first author, sample size, baseline severity of psoriasis, age, number of males and females, intervention (dosage and route), duration of treatment, mental health outcomes, psoriasis severity outcomes, adverse events (AEs), and safety.

## 3. Results

### 3.1. Systematic Review

A total of 803 records were identified via electronic database searches; 222 were removed as duplicates, resulting in 581 records for subsequent review. Of these studies, 556 were eliminated based on their titles and abstracts during the initial screening process. Among the 25 papers chosen for full‐text review, 16 were excluded for several reasons, leaving 9. Among the 4 articles that were found by citation searching, only one was eligible. Thus, the systematic review encompassed 10 studies.

### 3.2. Study Characteristics

Each of the 10 included studies investigated the effects of various psoriatic medications, primarily biologics, on mental health outcomes, with particular emphasis on depression and suicidal ideation (Table 1). The studies were published from 2014 to 2024, with the majority being randomized controlled trials (RCTs). Studies were conducted across multiple geographic locations, including the USA, the UK, and Japan. The majority of the studies examined patients with moderate‐to‐severe psoriasis and used various psoriatic treatments, including guselkumab, bimekizumab, brodalumab, and apremilast. Treatment durations ranged from short‐term (16 weeks) to long‐term follow‐ups (up to 5 years). Mental health outcomes were evaluated using instruments such as the hospital anxiety and depression scale (HADS) and the general health questionnaire (GHQ‐30), whereas psoriasis severity was frequently assessed with the psoriasis area and severity index (PASI) and minimal disease activity (MDA) criteria.

**Table 1:** Studies investigating the impact of psoriasis treatments on depression and suicide.

First author	Country	Study design	Number of patients	Baseline severity of Psoriasis	Age (mean)	Male/ female	Intervention (dosage and route)	Duration of treatment	Mental health outcomes	Psoriasis severity outcomes	Adverse events (AEs) and safety	Quality assessment
Gordon et al. [13]	Multiple countries	RCT	992 patients	Moderate‐to‐severe psoriasis	43.5 ± 12.2	692/300	Guselkumab (subcutaneously at a dosage of 100 mg at weeks 0, 4, 12, and 20), compared to placebo	24 weeks	By week 16, 59.2% of patients treated with guselkumab had HADS‐Depression levels below 8, considerably exceeding the 27.0% in the placebo group (*p* < 0.001), with additional improvement over adalimumab found by week 24	Significant improvements in PASI scores (reduction in psoriasis severity)	Common AEs included nasopharyngitis and upper respiratory tract infections	High
Blauvelt et al. [26]	USA	RCT	2480	Moderate‐to‐severe plaque psoriasis	N/A	N/A	Bimekizumab	Up to 5 years	Low rates of suicidal behavior are consistent with those observed in general psoriasis populations and other treatment modalities	High efficacy was noted with substantial skin clearance and maintenance over several years	TEAEs, such as minor infections, were reported, with a low incidence of suicidal ideation	Moderate
Lebwohl et al. [27]	USA	Cross‐sectional	4744	Moderate‐to‐severe plaque psoriasis	N/A	N/A	Brodalumab, administered via injection	5 years	During the 5 years, there were 54 reported cases of depression, with no new cases of suicide attempts or completed suicides	N/A	Common AEs included arthralgia, infections, some malignancies, and a few cardiovascular events	Moderate
Strober et al. [28]	USA	Prospective cohort	7490	Moderate‐to‐severe	48.06 ± 13.8	N/A	Biologics, conventional systemic therapies, and phototherapy, with assessments every 6 months	Several years	Lower rates for biologic‐treated patients (3.01 per 100 patient‐years) than phototherapy (5.85) and traditional systemic therapy (5.70). Biologics decreased depressed symptoms by 0.76 compared to conventional therapy. Conventional therapies	Moderate‐to‐severe cases managed with biologic and conventional therapies	AEs of depression were rare, with incidence rates of 0.21 per 100 patient‐years for biologics, and suicidality was low, with 21 cases across the study cohort of 7490	Moderate
Margolis et al. [29]	USA	Retrospective cohort	262,552	N/A	N/A	N/A	Biologic therapies, including adalimumab, etanercept, ustekinumab, ixekizumab, and secukinumab, were compared to methotrexate	Several years	Patients with psoriasis receiving biologic treatments had a lower incidence of mental disorders compared to those undergoing methotrexate treatment or not receiving biologic therapy	N/A	No increased risk of psychiatric illness, depression, or suicidality was associated with biologic therapies compared to nonbiologic treatments	High
Kavanaugh et al.[30]	USA	RCT	1493	Moderate‐to‐severe	N/A	N/A	Apremilast 30 mg or 20 mg, administered orally twice daily	5 years (260 weeks)	Depression rates were low (≤1.8%) across the study duration, with no significant increase in suicidal ideation or behavior	Sustained improvements in ACR20, ACR50, and ACR70 response rates, with 67.2% of patients achieving ACR20 at 5 years. Additionally, notable reductions were seen in swollen joint count, tender joint count, enthesitis, and dactylitis	Common AEs included diarrhea, nausea, headache, and respiratory infections	High
Rivera‐Oyola et al. [31]	USA	Case report series	3	Moderate‐to‐severe plaque psoriasis	28 to 63 years	2/1	Brodalumab	Case‐specific, ranging from months to recurrent follow‐ups	Two patients demonstrated improvements in depressive symptoms, whereas one patient presented with mixed psychiatric symptoms necessitating hospitalization	All three patients achieved PASI 100, indicating complete skin clearance after Brodalumab treatment	N/A	Low
Takahashi et al. [32]	Japan	Retrospective cohort	119	Moderate‐to‐severe	N/A	79/40	Biologics, systemic agents, or topical treatments	16 weeks	Biologic treatments resulted in a 52.2% decrease in GHQ‐30 scores, signifying a noticeable enhancement in mental health. Systemic treatments demonstrated a reduction of 23.9%, while topical treatments exhibited a reduction of 17.3%	Significant reduction in PASI scores across all treatment types, with biologics being the most effective	N/A	High
Yeroushalmi et al. [33]	USA	Cross‐sectional	Not specified	Moderate‐to‐severe	N/A	N/A	15 systemic agents for psoriasis, including four oral agents (acitretin, apremilast, methotrexate, and cyclosporine) and 11 biologics (e.g., adalimumab, etanercept, infliximab, certolizumab)	N/A	Rates of depression and suicidality linked to systemic therapies show that patients on oral agents report higher odds of depression compared to those on biologics	No direct measure of psoriasis severity change	Higher reporting odds of depression for oral agents, specific drugs like brodalumab and apremilast	Low
Coates et al.[34]	UK	RCT	739	Patients had active PsA	N/A	N/A	Guselkumab (100 mg is administered subcutaneously every 4 weeks or at weeks 0 and 4, then every 8 weeks.)	100 weeks	Depression and other mental health outcomes were indirectly improved as physical symptoms lessened, with over two‐thirds of patients achieving low levels of disease activity	~70% of patients achieved near remission in swollen joints, PASI, and enthesitis through week 100	N/A	High

*Note:* This table presents key details of the included studies assessing the impact of psoriasis treatments on depression and suicide. It summarizes study characteristics, treatment interventions, patient demographics, psoriasis severity, mental health effects, and safety profiles.

Abbreviations: ACR, American College of Rheumatology (ACR) Response Criteria; AE, adverse event; GHQ‐30, general health questionnaire (30‐item version); HADS, hospital anxiety and depression scale; PASI, psoriasis area and severity index; PsA, psoriatic arthritis; RCT, randomized controlled trial; TEAEs, treatment‐emergent adverse events.

### 3.3. Depression Outcomes

Based on these studies, the impact of psoriatic treatments on depression generally suggests that biologic therapies, particularly guselkumab, brodalumab, and bimekizumab, reduce depressive symptoms in psoriasis patients. For instance, Gordon et al. [13] found that by week 16, guselkumab significantly improved depression symptoms and PASI scores in psoriasis patients compared to placebo, especially in those with baseline moderate‐to‐severe depressed symptoms. Patients treated with guselkumab showed sustained and significant reductions in depression symptoms and PASI scores by week 24 (*p*  < 0.0001) [13]. Similarly, Coates et al. [34] demonstrated that guselkumab significantly alleviated joint and skin symptoms in patients with active psoriatic arthritis (PsA). Their analysis demonstrated that physical improvements over time led to a substantial reduction in the overall disease burden, with over half of the patients successfully achieving the composite MDA threshold by week 100 [34].

Rivera‐Oyola et al. [31] demonstrate that brodalumab resulted in complete skin clearance (PASI 100) in three patients with psoriasis and psychiatric conditions, including those with previous treatment resistance. Two of the three patients exhibited a decrease in depressive symptoms following treatment. One patient necessitated psychiatric hospitalization due to a combination of acute manic episodes, psychotic features, and dangerous impulsive behavior. However, the patient denied any suicidal ideation, and no direct causal relationship between brodalumab and increased self‐injurious behavior was established [31].

Kavanaugh et al. [30] demonstrated that apremilast sustained its efficacy over a 5‐year period of follow‐up, significantly alleviating symptoms of PsA without an associated increase in depression or suicidal ideation. Throughout the study, depression rates remained low (≤1.8%), and no new psychiatric safety concerns were identified with prolonged treatment [30].

Research indicates that patients receiving biologic medications experienced lower rates of depression compared to those receiving conventional treatments, indicating a mental health benefit associated with biologic therapies. They also claimed that certain biologics may improve quality‐of‐life, correlating with a reduction in depressive symptoms, as demonstrated by lower scores on the GHQ‐30 and the Dermatological Life Quality Index [32].

Margolis et al. [29] utilized electronic medical records to directly compare biologics with methotrexate, a primary first‐line systemic medication. Margolis et al. [29] demonstrated that psoriasis patients receiving biologic medications exhibited a markedly reduced risk of developing psychiatric illnesses (hazard ratio 0.52) in comparison to those not receiving such treatments. The study indicated that patients receiving biologic treatments had a lower likelihood of being diagnosed with depression and suicidality compared to those undergoing traditional therapies such as methotrexate [29].

Phototherapy was evaluated among the reviewed treatment modalities for patients with psoriasis; however, its effect on comorbid depression showed less favorable outcomes compared to targeted systemic therapies. Strober et al. [28] demonstrated that biologic medications markedly reduced the incidence of depressive symptoms, with a rate of 3.01 per 100 patient‐years, compared with 5.85 per 100 patient‐years for phototherapy and 5.70 per 100 patient‐years for conventional systemic treatments. The hazard ratio for developing depression was 0.76 using biologics compared with the conventional therapy group as the reference [28].

Regarding conventional first‐line systemic immunomodulatory therapies, Yeroushalmi et al. [33] conducted a comprehensive postmarketing analysis of conventional oral systemic medicines such as methotrexate, acitretin, and cyclosporine. They reported that Brodalumab and apremilast had the highest reporting odds ratios (RORs) for depression. Apremilast showed a strong psychiatric effect. Psoriasis patients on apremilast demonstrated the highest ROR for depression at 4.88 (*p*  < 0.001), followed by Brodalumab with the second‐highest ROR of 4.08 (*p*  < 0.001). After excluding brodalumab and apremilast, oral agents (acitretin, methotrexate, and cyclosporine) had a higher ROR than biologics (OR = 2.42). Among biologics, TNF‐alpha inhibitors (certolizumab, infliximab, and adalimumab) had higher RORs, while etanercept, secukinumab, and ixekizumab had the lowest. Adalimumab, a commonly prescribed first‐line biologic, demonstrated an initial favorable ROR of 0.59, which shifted to 1.23 after accounting for the reporting bias. Conversely, the conventional first‐line oral medication, methotrexate, had a significantly increased probability of reported depression after accounting for reporting bias (ROR = 2.00), which the authors indicate may be exacerbated by its established side effect profile, including fatigue [33].

Across the included studies, biologic therapies were the most frequently examined treatment category. Evidence for conventional systemic agents, including methotrexate, cyclosporine, and acitretin, was less detailed and was mainly derived from comparative or pharmacovigilance analyses rather than direct treatment‐specific evaluations of depression outcomes.

### 3.4. Suicide Ideation Outcomes

The evaluation of suicidal ideation reveals mixed findings, indicating that most biologics present minimal risk; however, caution is warranted with specific medications. For instance, the 5‐year pharmacovigilance report by Lebwohl et al. [27] evaluated the long‐term safety profile of brodalumab, an IL‐17 receptor antagonist, for moderate‐to‐severe plaque psoriasis. Although brodalumab carries a boxed warning for suicidal ideation and behavior (SIB), no causal relationship has been established. 54 occurrences of depression were observed during 5 years; by year five, the incidence dropped from 1.29 to 1.14 incidents per 100 patients. Importantly, regarding severe psychiatric events, no completed suicides were recorded throughout the 5 years of the study, and no new suicide attempts were reported during the fifth year [27].

Blauvelt et al. [26] reported that the incidence of SIB with bimekizumab was 0.13 per 100 patient‐years, comparable to rates observed in the general psoriasis population and other IL‐17A and IL‐23 biologics. The incidence of depression was low, with 92.9% of bimekizumab‐treated patients experiencing no or minimal depression symptoms by week 16, in contrast to 81.1% in the placebo group. This study indicates that bimekizumab does not elevate the risk of depression or suicidality and demonstrates safety comparable to other biologics regarding mental health outcomes [26]. These findings indicate that although some biologics have precautionary warnings, careful monitoring and appropriate patient selection can effectively manage psoriasis with minimal adverse mental health effects, particularly regarding suicidal ideation.

## 4. Discussion

Effective management of psoriasis patients involves more than merely selecting and prescribing the appropriate medication. Effective management of chronic diseases requires a comprehensive strategy that covers patient education, screening for comorbidities, and therapy adjustments based on alterations in clinical manifestations [19]. Psoriasis impacts not only the skin of the patient but also adversely influences self‐esteem, social life, and overall quality‐of‐life [35] by causing anxiety, depression, and isolation. Psoriasis patients with anxiety or depression may incur additional treatment costs associated with their psychiatric disorder [13, 36]. Psychosocial stress may both cause and worsen psoriasis. Thus, psoriasis might be called a psychosocial skin condition [14]. Studies reported that patients with psoriasis, and especially PsA, have an elevated risk of developing depression and self‐injurious behavior. Patients frequently experience stigma associated with psoriasis and commonly report feelings of rejection and shame. Isolation and feelings of hopelessness significantly burden individuals affected by the condition [31]. Due to the chronic nature of psoriasis, depression and suicidal thoughts may exacerbate over time. Even when clinical symptoms improve significantly, rehabilitation from mental illnesses might take longer than anticipated due to cumulative consequences [13].

A recent study examined the annual incidence rates of depression, suicidal ideation, and suicide attempts among patients with psoriasis and patients with PsA. The incidence of depression was higher in both groups relative to the general population [37]. It is worth noting that socioeconomic factors emerged as significant considerations. The analysis indicates that attaining a higher level of education is associated with a decreased risk of developing depressive symptoms [38]. Also, having private health insurance was associated with a reduced risk of developing depressive symptoms [28]. A recent meta‐analysis by Singh et al. [39], including 330,207 individuals with psoriasis, indicated a higher likelihood of suicidal ideation among those affected by the condition.

The question that arises is whether the aforementioned symptoms can be improved by the timely and appropriate treatment of psoriasis and, conversely, whether these therapeutic options can cause adverse effects such as depression and suicidal ideation. While psoriasis hugely impacts patients’ quality‐of‐life [40], a definite cure remains elusive. Management strategies for psoriasis currently focus on symptom alleviation, quality‐of‐life enhancement, and disease progression prevention [41]. Topical therapies, phototherapy, oral systemic therapies, and biologic therapies represent standard treatment modalities for psoriasis [42]. These treatments demonstrate a range of side effects that impact patient satisfaction, including increased skin atrophy, irritant contact dermatitis, and potential systemic complications such as headaches [18, 43].

In addition to the psychosocial effects on psychopathology, it would not be surprising if psoriasis had a biological connection to brain function given the fact that it is an inflammatory immune dysfunction and that inflammation and immune function have known effects on the brain. Interestingly, the skin and brain both develop from the same embryonic ectoderm. Emerging theories suggest a connection between depression and inflammation. Elevated levels of various cytokines, such as IL‐1, IL‐6, and tumor necrosis factor‐alpha, pivotal in psoriasis, have been linked to depression [44]. Consequently, reducing their levels may mitigate the impact of these cytokines on depressive symptoms. Biologic treatments targeting specific cytokines, such as IL‐23 blockade with guselkumab, have demonstrated improvements in psoriasis that correlate with reductions in anxiety and depression. Nevertheless, it is challenging to determine the extent to which the improvements in skin disease are directly attributable to the improvements in anxiety and depression and to what extent they are independent effects [13].

The visibility of psoriasis lesions can result in social stigmatization and subsequent depression. Hence, improvement in psoriasis clinical symptoms is likely to enhance social functioning and quality‐of‐life, potentially reducing the risk of developing depressive symptoms and depression [28]. Biologic therapies are linked to a low risk of suicidality; however, previous observations indicate an imbalance in suicidal and suicidal ideation among patients receiving these treatments. Causality remains uncertain, and variations in the study design may contribute to the observed imbalance. It is important to note that treatment adherence varies across studies, leading to different results.

Considering the role of TNF‐α in depression, further research may be advantageous to ascertain whether biologic therapies targeting other cytokines exert a different influence on depression and suicide compared to anti‐TNF‐α agents [28]. Individuals prescribed any biologic or a specific biologic agent were generally and consistently less likely to receive a psychiatric illness diagnosis compared to those prescribed methotrexate, except for suicidal ideation and suicide. No significant differences were observed between therapies regarding suicidal ideation and suicidal outcomes. The articles showed that biologics can improve the patient’s clinical symptoms, and this improvement in clinical symptoms can also result in an improvement of their mood and psychological condition [29].

These findings suggest that systemic psoriasis therapies should not be interpreted as a single homogeneous category when considering psychiatric outcomes. Biologic therapies had the strongest evidence for improvement or stability of depressive symptoms, whereas the evidence for conventional systemic agents such as methotrexate, cyclosporine, and acitretin was more limited. Brodalumab and apremilast require cautious interpretation because depression‐ or suicidality‐related signals have been reported in some safety and pharmacovigilance data, although causality has not been established. Therefore, in patients with active depression, previous suicidal ideation, or other psychiatric comorbidities, systemic treatment selection should include individualized risk–benefit assessment, baseline psychiatric screening, and collaboration between dermatologists and mental health professionals. Evidence regarding phototherapy and psychiatric outcomes remains limited. Phototherapy is generally considered when topical therapy is insufficient and may be used in patients with more extensive or moderate‐to‐severe psoriasis [16, 19]. In the included studies, phototherapy was not extensively evaluated as an independent intervention for depression, anxiety, or suicidal ideation. Available evidence suggests that phototherapy can improve psoriasis severity and quality‐of‐life, but its independent effect on psychiatric outcomes remains unclear [28]. They also showed that depressive symptom rates were higher among patients receiving phototherapy than among those receiving biologic therapy. However, this finding should be interpreted cautiously because treatment selection may reflect differences in disease severity, previous treatment exposure, access to biologic therapy, comorbidities, and other patient‐level factors [28]. Therefore, current evidence is insufficient to conclude that phototherapy independently reduces depression or suicide risk in patients with psoriasis.

### 4.1. Limitations

Our study presented certain limitations. Initially, most of our inclusion studies were conducted in the USA, potentially influencing the results of the study. We advocate for similar research in other countries. Second, most of the patients included in our study exhibited moderate‐to‐severe disease severity. Different results may be observed in patients exhibiting more or fewer joint or skin symptoms.

## 5. Conclusion

This review explores the strong link between psoriasis, depression, and suicidal thoughts and discusses how various treatments can impact these challenges. Biologic therapies seem promising for helping with the emotional challenges of psoriasis, giving hope to many patients. Still, the findings on suicidal thoughts are varied, highlighting that each patient’s experience is different. Treating psoriasis involves more than just focusing on the skin; it’s important to consider a holistic approach. Working together, dermatologists and mental health professionals can make sure that patients receive the complete care they need. In the future, larger studies could help us grasp these connections and enhance the quality‐of‐life for people with psoriasis.

## Funding

The authors declare that this research did not receive any specific grant from funding agencies.

## Conflicts of Interest

The authors declare no conflicts of interest.

## Data Availability

I claim that all the data supporting the findings of this study are contained within the article and its accompanying tables.
